# Evaluation of Psychometric Properties of Thai Version Telehealth Usability Questionnaire (T-TUQ)

**DOI:** 10.5195/ijt.2023.6577

**Published:** 2023-12-12

**Authors:** Parima Hirunwiwatkul, Punnaka Pongpanich, Wasee Tulvatana, Supharat Jariyakosol, Warongporn Phuenpathom, Supaporn Krittanupong, Ruttanabhorn Chonramak, Tidarat Pichedvanichok, Roongroj Bhidayasiri, Chaichana Nimnuan

**Affiliations:** 1 Department of Ophthalmology, King Chulalongkorn Memorial Hospital (KCMH), Bangkok, Thailand; 2 Department of Ophthalmology, Faculty of Medicine, Chulalongkorn University, Bangkok, Thailand; 3 School of Medicine, Mae Fah Luang University, Chiang Rai Province, Thailand; 4 Chulalongkorn Centre of Excellence for Parkinson's Disease & Related Disorders, Department of Medicine, Faculty of Medicine, Chulalongkorn University and KCMH, Thai Red Cross Society, Bangkok, Thailand; 5 Chula Neuroscience Center, KCMH, Thai Red Cross Society, Bangkok, Thailand; 6 Nursing Department, KCMH, Bangkok, Thailand; 7 The Academy of Science, The Royal Society of Thailand, Bangkok, Thailand.; 8 Department of Psychiatry, Faculty of Medicine, Chulalongkorn University, Bangkok, Thailand

**Keywords:** Questionnaire, Telehealth, Telehealth Usability Questionnaire (TUQ), Thai, Translation, Usability, Validation

## Abstract

This cross-sectional validation study aimed to translate, cross-culturally adapt, and investigate the psychometric properties of a Thai version of the Telehealth Usability Questionnaire (T-TUQ). Two hundred and ten Thai participants, mean age of 61.2±15.2 years, were recruited from three specialty clinics: 50 (23.8%) hematology, 70 (33.3%) movement disorders, and 90 (42.9%) general neurology. The T-TUQ was translated from the original English version to produce a Thai language version. Back translation and pilot cognitive interviews were completed. All five subscales (usefulness, ease of use, effectiveness, reliability, and satisfaction) showed excellent internal consistency (alpha >0.80), displayed by Cronbach's alpha coefficient of 0.83, 0.94, 0.86, 0.83, and 0.92, respectively. For construct validity, exploratory factor analysis revealed two dimensions from eigenvalues and scree plot, defined as utility and accessibility subscales. In conclusion, the T-TUQ could be a reliable and valid instrument to evaluate the usability of telehealth with a Thai population.

Since the onset of the Coronavirus Disease 2019 (COVID-19) pandemic, telehealth implementation has expanded globally to provide remote access to healthcare and to aid social distancing efforts (Grossman et al., 2020; Lai et al., 2020). Since the early 2000s, many questionnaires have been constructed to explore the benefits and disadvantages of telehealth. These questionnaires gathered feedback from users on different aspects of telehealth implementation including effectiveness, convenience, satisfaction, and acceptance in order to optimize future telehealth services (Barsom et al., 2020; Hajesmaeel-Gohari & Bahaadinbeigy, 2021; Kruse et al., 2017; Langbecker et al., 2017; Parmanto et al., 2016; Weaver et al., 2021).

The Telehealth Usability Questionnaire (TUQ) has been reported to be one of the most commonly used surveys to evaluate the usability of telehealth services in various medical fields (Finn et al., 2021; Hajesmaeel-Gohari & Bahaadinbeigy, 2021; Park et al., 2021; Schutte et al., 2012). The questionnaire was designed to cover all five aspects of usability by combining factors from pre-existing validated telehealth and general system usability questionnaires (Parmanto et al., 2016). Good to excellent content reliability from 53 participants, mainly Caucasians, of either patients or clinicians was reported for all constructs. Due to its reliability, acceptance and comprehensiveness, TUQ is considered an effective instrument to measure telehealth usability (Barsom et al., 2020; Hajesmaeel-Gohari & Bahaadinbeigy, 2021; Langbecker et al., 2017; Parmanto et al., 2016; Weaver et al., 2021).

Studies have reported the use of non-English language versions of the TUQ; however, the TUQ has never been translated or officially applied in Thailand (Bibiloni et al., 2020; Hvalič Touzery et al., 2020). The application of the original TUQ in Thailand would be limited because of the language barriers and cultural difference. Moreover, the objective of this study was to develop and validate a Thai version of the Telehealth Usability Questionnaire (T-TUQ) focusing on the patients' perspectives, and not including the clinicians' perspective.

## Materials and Methods

This cross-sectional validation study was approved by the Institutional Review Board of the Faculty of Medicine, Chulalongkorn University (Certificate of approval number 1077/2021). The study was carried out in compliance with the Declaration of Helsinki. All participants were thoroughly informed about the study before providing a consent by action. Developers of the original TUQ gave their permission to translate the questionnaire into Thai without fee (Parmanto et al., 2016).

### Participants

Inclusion criteria were native Thai speakers aged 18 years or older with at least one experience of a telehealth visit from the hematology, movement disorder, or general neurology clinics of King Chulalongkorn Memorial Hospital (KCMH), Thailand, and the ability to use the hospital's telehealth application either by themselves or with the help of their caregivers. Eligible participants were recruited between July 16, 2021 and November 10, 2022 within one month of their telehealth visit. Participants who refused to complete the questionnaire were excluded.

A total of 210 Thai participants, mean age of 61.2±15.2 years, were recruited from three specialty clinics: 50 (23.8%) participants with hematologic conditions from hematology, 70 (33.3%) dystonia participants from movement disorders, and 90 (42.9%) participants with other neurological conditions such as facial dystonia, stroke, or seizure from general neurology. Nearly half of the participants completed the questionnaire on their own (n=95, 45.2%), while the remaining subjects were interviewed (n=115, 54.8%).

### Instrument

Twenty-one items in the original TUQ were designed to comprehensively evaluate five usability factors of telehealth systems: usefulness, ease of use, effectiveness, reliability, and satisfaction. Usability is calculated by averaging the score of all items, rated by a 7-point Likert scale ranging from 1 (strongly disagree) to 7 (strongly agree). The TUQ was incorporated from three existing questionnaires: Telemedicine Satisfaction Questionnaire (TSQ), Telemedicine Patient Questionnaire (TMPQ), and Telemedicine Satisfaction and Usefulness Questionnaire (TSUQ). These questionnaires were widely used with proper content validity, but only partially cover the usability factors. Data for the TUQ content reliability, displayed in Table 1, was collected from self-reporting 53 participants of either patients or clinicians. The ethnicity of the TUQ's participants were 42 (79.2%) Caucasian, 5 (9.5%) Asian, 3 (5.6%) African American, 2 (3.7%) Hispanic, and 1 (2%) other (Parmanto et al., 2016). In this study, the T-TUQ responses were collected either by interview or self-reported, according to the participant's preference.

**Table 1.** T-TUQ Subscales Usability Score and Cronbach's Alpha Compared with Original TUQ

Subscales (N of T-TUQ)	Mean Score (SD)	Cronbach's alpha			
		T-TUQ		Original TUQ	
		Raw	Standardized	Raw	Standardized
Usefulness (209)	6.61 (0.81)	0.83	0.84	0.83	0.85
Ease of use (208)	6.51 (0.91)	0.94	0.94	0.92	0.93
Effectiveness (209)	6.51 (0.93)	0.86	0.86	0.86	0.87
Reliability (172)	6.15 (1.27)	0.83	0.84	0.79	0.81
Satisfaction (205)	6.64 (0.81)	0.92	0.93	0.91	0.92

*Note*. N = Valid cases per subscales for reliability analysis, SD = Standard deviation, T-TUQ = Thai version telehealth usability questionnaire, TUQ = Telehealth usability questionnaire

### Translation and Adaption Process

The original English version of the TUQ was translated into Thai in accordance with the guidelines for the process of cross-cultural adaptation of self-report measures provided by Beaton et al. (2000). Forward and backward translations were conducted by native Thai-speaking and native English-speaking translators, respectively. Discussions with an expert committee were made throughout the process for proper cross-cultural adaptation. The main discussions were about the proper Thai word for “system” and “productive” in the original version. The back-translated TUQ version was later compared with the original TUQ for linguistic and conceptual accuracy. A pilot face validation was then carried out with ten native Thai-speaking participants from the general neurology clinic. These pilot participants were not included in the study sample. No changes were made for the final version as the pilot test reported the T-TUQ to be fully comprehensible. The final T-TUQ is available in the Appendix.

### Sample Size Calculations

Comrey (1988) and Guadagnoli and Velicer (1988) suggested a sample size of 200 to 300 for factor analysis. Conforming to the rule of thumb for questionnaire psychometric properties testing, one question required 10 samples (Nunnally, 1978). Considering the 21 items in the TUQ, the targeted sample size was 210 with no drop-out expected.

### Statistical Analysis

All analyses were performed using Microsoft Excel and SPSS for Windows (version 28.0; IBM Corp) without imputation of missing data.

### Descriptive Analysis

Continuous variables were displayed by mean with standard deviation (SD). Frequency with percentages were used to present categorical variables.

### Reliability

Internal consistency within each subscale was determined by Cronbach's alpha coefficient. An alpha of at least 0.8 was considered as good consistency (Portney & Watkins, 2009).

### Validity

Content validity remained from the original version due to adherence to the previously mentioned translation and adaptation process. Construct validity was assessed by exploratory factor analysis using principal axis factoring (PAF) with Oblimin and Kaiser normalization rotation methods. The factorability of the sample matrix was measured by the Kaiser-Meyer-Olkin (KMO) test requiring a minimum acceptable value of 0.6 (Kaiser, 1974). Bartlett's test of sphericity was applied to assess the correlation matrix.

## Results

From 210 T-TUQ responses of Thai participants with neurological or hematological disorders, the average summary score of Thai version TUQ was 6.5 (SD 0.95) out of 7. High mean usability scores were reported for all five subscales (Table 1).

### Reliability

In terms of comparison with the original TUQ with five subscales, the T-TUQ (usefulness, ease of use, effectiveness, reliability, and satisfaction) had good to excellent internal consistency displayed by Cronbach's alpha of 0.83, 0.94, 0.86, 0.83, and 0.92, respectively (Table 1).

### Constructed Validity

**Figure 1.**
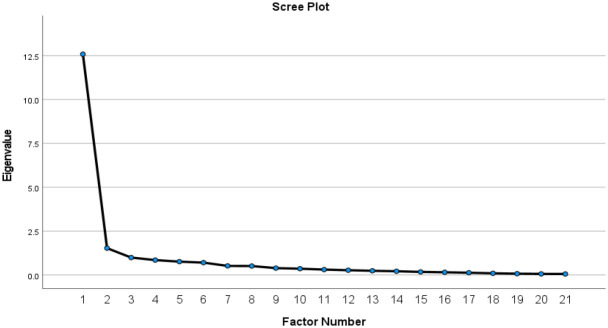
Scree Plot of Thai Version Telehealth Usability Questionnaire Exploratory Factor Analysis

Since the KMO index was 0.91 and the Bartlett's test of sphericity was significant (< 0.001), this matrix of samples was deemed adequate for factor analysis. Exploratory factor analysis by PAF was done to determine the number of factors and the degree they were related. Rising the iteration to 9999 attempts to extract 5 factors as the original TUQ was terminated because the communality of a variable exceeded 1.0. While one factor extracted with eigenvalues >1 was responsible for 58.3% of variance, a two-factor structure explained a total of 63.9% of variance. Supported by the scree plot in Figure 1, two-factor structure was later evaluated by content. Nine items in the T-TUQ: items 4, 9, 16, 17, 5, 7, 10, 14, and 6, were loaded into factor 2, which can be defined as the telehealth accessibility subscale (Table 2). Twelve other items in factor 1 were defined as the utility subscale. High factor correlation of 0.76 was reported between two factors. Considering the two-factor constructs, internal consistency using Cronbach's alpha was 0.95 for both factors.

**Table 2.** Factor Loadings of the Thai Version Telehealth Usability Questionnaire (n=210)

	Factor	Factor
	1	2
Item 19	0.928	
Item 18	0.922	
Item 1	0.911	
Item 20	0.758	
Item 8	0.736	
Item 2	0.697	
Item 21	0.676	
Item 15	0.671	
Item 12	0.669	
Item 11	0.668	
Item 3	0.618	
Item 13	0.504	
Item 4		0.865
Item 9		0.844
Item 16		0.833
Item 17		0.812
Item 5		0.756
Item 7		0.683
Item 10		0.614
Item 14		0.593
Item 6	0.345	0.494

*Note*. Extraction Method: Principal Axis Factoring, Rotation Method: Oblimin with Kaiser Normalization

## Discussion

The primary objective of this study was to translate, cross-culturally adapt, and assess the psychometric properties of a Thai version of the TUQ. The face validation process, along with the responses from 210 native Thai-speaking participants, confirmed that the T-TUQ was easily readable and exhibited content validity equivalent to the original English version. Furthermore, the T-TUQ demonstrated good to excellent reliability.

Before the onset of the COVID-19 pandemic, the TUQ gained recognition for its popularity, reliability, and comprehensiveness. With the global increase in telehealth services, the original questionnaire was translated into various languages. These translations extended to Spanish, Turkish, Slovene, Urdu, Portuguese, and Danish, aiming to capture user feedback on emerging telehealth technologies. In the context of Thailand, where there was no standard telehealth-related questionnaire in Thai, the development of a reliable T-TUQ would serve as an essential tool to enhance the implementation of telehealth services.

The internal consistencies of all five T-TUQ subscales displayed good to excellent reliability, aligning with reports from the original TUQ by Parmanto et al. (2016) (Table 1). A higher Cronbach's alpha of greater than 0.9 was reported in the Turkish version (n=107) (Özkeskin et al., 2021), but lower values of reliability (0.24) and usefulness (0.58) were reported from the Danish TUQ (n=34) (Bender et al., 2022). The Brazilian TUQ (n=64) also demonstrated excellent internal consistency (alpha=0.94) (Santos et al., 2023). Translations into Spanish and Slovene languages were conducted, but no reliability details have been reported. Our study, which included participants from hematology and neurology clinics, supports greater generalizability of the T-TUQ due to the range of diseases included. This study boasted the second-largest sample size, following the Urdu study that involved 350 participants. The analysis of construct validity demonstrated two-factor constructs and exhibited excellent internal consistency for both constructs. As the attempt to extract five factors as the original TUQ was terminated, this study data did not yet support five subscales. These results supported a reduction in the T-TUQ subscales from five to two. The reduced subscales were titled “Accessibility” and “Utility.” In comparison to the original TUQ, the “Accessibility” subscale was composed of items from the “Usefulness,” “Ease of use” (except item 8). “Effectiveness” (except items 13 and 14), while “Reliability” (except item 15) and “Satisfaction” were grouped under the “Utility” subscales (Table 3). However, further validation through confirmatory factor analysis should be conducted to support the T-TUQ. This reduction in factor constructs could be attributed to cultural and language differences. In the Thai language, items in factor 2 or “Utility” pertain more to feelings and inner self-benefit, whereas items in factor 1 are more related to the operating system and processes. Additionally, the high factor correlation of 0.76 between the two constructs and the scree plot in Figure 1 indicated that a one-factor structure could also be considered.

**Table 3.** Five-factor Structure Compared with Two-factor Structure Usability Subscales

Five-factor Structure Subscales	Questionnaire Items	Two-factor structure subscales
Usefulness		
1	Telehealth improves my access to healthcare services	Accessibility
2	Telehealth saves me time traveling to a hospital or specialist clinic	Accessibility
3	Telehealth provides for my healthcare needs	Accessibility
Ease of Use		
4	It was simple to use this system	Accessibility
5	It was easy to learn to use the system	Accessibility
6	I believe I could become productive quickly using this system	Accessibility
7	The way I interact with this system is pleasant	Accessibility
8	I like using the system	Utility
9	The system is simple and easy to understand	Accessibility
Effectiveness		
10	This system is able to do everything I would want it to be able to do	Accessibility
11	I could easily talk to the clinician using the telehealth system	Accessibility
12	I could hear the clinician clearly using the telehealth system	Accessibility
13	I felt I was able to express myself effectively	Utility
14	Using the telehealth system, I could see the clinician as well as if we met in person	Utility
Reliability		
15	I think the visits provided over the telehealth system are the same as in-person visits	Accessibility
16	Whenever I made a mistake using the system, I could recover easily and quickly	Utility
17	The system gave error messages that clearly told me how to fix problems	Utility
Satisfaction		
18	I feel comfortable communicating with the clinician using the telehealth system	Utility
19	Telehealth is an acceptable way to receive healthcare services	Utility
20	I would use telehealth services again	Utility
21	Overall, I am satisfied with this telehealth system	Utility

This study has limitations, including the restricted generalizability of the T-TUQ due to its exclusive focus on participants from hematology and neurology clinics at a university hospital in Bangkok. Moreover, test and re-test processes were not conducted, and the construct validation was carried out through exploratory, rather than confirmatory, factor analysis.

Future studies could expand participant characteristics to include individuals from other regions in Thailand with various diseases. Additionally, conducting test and re-test processes and confirmatory factor analysis would enhance the reliability and validity of the T-TUQ.

## Conclusion

The development and validation of the Thai version of the Telehealth Usability Questionnaire (T-TUQ) represent a significant step toward evaluating telehealth services in Thailand. While the initial findings on its reliability and validity show promise, further research is needed to confirm its utility across diverse medical specialties and patient populations. The two-factor structure observed in the T-TUQ suggests potential cultural and language influences on user perceptions of telehealth. Future studies should expand the participant pool, conduct test-retest processes, and employ confirmatory factor analysis to enhance the questionnaire's reliability and validity. Ultimately, a validated T-TUQ holds great potential for improving telehealth implementation in Thailand, facilitating patient-centered care, and ensuring technology effectively serves its users.

## Authors Note

No author has any financial or other conflicts of interest to disclose. We thank Teerakiat Jaroensettasin, MD, consultant in Child and Adolescent Psychiatry, Colchester, UK, and his team for the back-translation process. For permission to translate the Telehealth Usability Questionnaire (TUQ), we thank Bambang Parmanto, PhD., professor and chair Department of Health Information Management Department, School of Health and Rehabilitation Sciences, University of Pittsburgh, Pennsylvania, U.S., and his team. Parima Hirunwiwatkul, MD and Punnaka Pongpanich, MD contributed to the work equally as co-first authors.
