# Corrections to: Hirunwiwatkul, P., Pongpanich, P., Tulvatana, W., Jariyakosol, S., Phuenpathom, W., Krittanupong, S., Chonramak, R., Pichedvanichok, T., Bhidayasiri, R., Nimnuan, C. (2023). Evaluation of psychometric properties of Thai version Telehealth Usability Questionnaire (T-TUQ), *International Journal of Telerehabilitation*, *15*(2), 1-14. https://doi.org/10.5195/ijt.2023.6577

**DOI:** 10.5195/ijt.2024.6633

**Published:** 2024-06-28

**Authors:** Parima Hirunwiwatkul

**Affiliations:** 1 Department of Ophthalmology, King Chulalongkorn Memorial Hospital (KCMH), Bangkok, Thailand; 2 Department of Ophthalmology, Faculty of Medicine, Chulalongkorn University, Bangkok, Thailand

## Abstract

The errors occurred by mixing the items of structure subscales (both in Table 3 and the text in the Discussion), and by adding in the Authors' Note, the name and Orchid number of a person not listed as an author. Changes have been made to correct these errors. The original article can be found via the DOI https://doi.org/10.5195/ijt.2023.6577

## Erratum

Page 6: Sentences in the Discussion were changed from: In comparison to the original TUQ, the “Accessibility” subscale was composed of items from the “Usefulness,” “Ease of use” (except item 8). “Effectiveness” (except items 13 and 14), while “Reliability” (except item 15) and “Satisfaction” were grouped under the “Utility” subscales (Table 3). However, further validation through confirmatory factor analysis should be conducted to support the T-TUQ. This reduction in factor constructs could be attributed to cultural and language differences. In the Thai language, items in factor 2 or “Utility” pertain more to feelings and inner self-benefit, whereas items in factor 1 are more related to the operating system and processes.

To: In comparison to the original TUQ, the “Accessibility” subscale was composed of items from the “Ease of use” (except item 8), and “Reliability” (except items 15); while “Usefulness”, “Effectiveness” (except items 10 and 14), and “Satisfaction” were grouped under the “Utility subscales (Table 3). However, further validation through confirmatory factor analysis should be conducted to support the T-TUQ. This reduction in factor constructs could be attributed to cultural and language differences. In the Thai language, items in factor 1 or “Utility” pertain more to feelings and inner self-benefit, whereas items in factor 2 are more related to the operating system and processes.

Page 7: Table 3 was changed from:

**Table 3:** Five-factor Structure Compared with Two-factor Structure Usability Subscales

Five-factor Structure Subscales	Questionnaire Items	Two-factor structure subscales
Usefulness		
1	Telehealth improves my access to healthcare services	Accessibility
2	Telehealth saves me time traveling to a hospital or specialist clinic	Accessibility
3	Telehealth provides for my healthcare needs	Accessibility
Ease of Use		
4	It was simple to use this system	Accessibility
5	It was easy to learn to use the system	Accessibility
6	I believe I could become productive quickly using this system	Accessibility
7	The way I interact with this system is pleasant	Accessibility
8	I like using the system	Utility
9	The system is simple and easy to understand	Accessibility
Effectiveness		
10	This system is able to do everything I would want it to be able to do	Accessibility
11	I could easily talk to the clinician using the telehealth system	Accessibility
12	I could hear the clinician clearly using the telehealth system	Accessibility
13	I felt I was able to express myself effectively	Utility
14	Using the telehealth system, I could see the clinician as well as if we met in person	Utility
Reliability		
15	I think the visits provided over the telehealth system are the same as in-person visits	Accessibility
16	Whenever I made a mistake using the system, I could recover easily and quickly	Utility
17	The system gave error messages that clearly told me how to fix problems	Utility
Satisfaction		
18	I feel comfortable communicating with the clinician using the telehealth system	Utility
19	Telehealth is an acceptable way to receive healthcare services	Utility
20	I would use telehealth services again	Utility
21	Overall, I am satisfied with this telehealth system	Utility

To:

**Table 3:** Five-factor Structure Compared with Two-factor Structure Usability Subscales

Five-factor Structure Subscales	Questionnaire Items	Two-factor structure subscales
Usefulness		
1	Telehealth improves my access to healthcare services	Utility
2	Telehealth saves me time traveling to a hospital or specialist clinic	Utility
3	Telehealth provides for my healthcare needs	Utility
Ease of Use		
4	It was simple to use this system	Accessibility
5	It was easy to learn to use the system	Accessibility
6	I believe I could become productive quickly using this system	Accessibility
7	The way I interact with this system is pleasant	Accessibility
8	I like using the system	Utility
9	The system is simple and easy to understand	Accessibility
Effectiveness		
10	This system is able to do everything I would want it to be able to do	Accessibility
11	I could easily talk to the clinician using the telehealth system	Utility
12	I could hear the clinician clearly using the telehealth system	Utility
13	I felt I was able to express myself effectively	Utility
14	Using the telehealth system, I could see the clinician as well as if we met in person	Accessibility
Reliability		
15	I think the visits provided over the telehealth system are the same as in-person visits	Utility
16	Whenever I made a mistake using the system, I could recover easily and quickly	Accessibility
17	The system gave error messages that clearly told me how to fix problems	Accessibility
Satisfaction		
18	I feel comfortable communicating with the clinician using the telehealth system	Utility
19	Telehealth is an acceptable way to receive healthcare services	Utility
20	I would use telehealth services again	Utility
21	Overall, I am satisfied with this telehealth system	Utility

Page 8: Person listed in Author note with ORCID ID who was not an author was removed from the author note: Buravej Assavapongpaiboon (https://orcid.org/0000-0002-5902-2162)

## Full Text Original Article

https://doi.org/10.5195/ijt.2023.6577

